# New insights into the genomic information of an overlooked human pathogen: *Bartonella rochalimae* causative agent of Carrion’s disease

**DOI:** 10.1371/journal.pntd.0013040

**Published:** 2025-04-17

**Authors:** Giovanna Mendoza-Mujica, Karen Daphne Calvay-Sanchez, Yanina Zarate-Sulca, Victor Jimenez-Vasquez

**Affiliations:** Laboratory of Vector-Borne Bacterial Diseases, National Institute of Health, Lima, Peru; Pasteur Institute of Iran, IRAN, ISLAMIC REPUBLIC OF

## Abstract

The *Bartonella* genus includes over twenty species, most transmitted by arthropods and possibly eleven related to human diseases, though some currently lack specific vectors or hosts. *Bartonella rochalimae*, a Gram-negative pleomorphic bacterium, was first isolated in 2007 from a woman who traveled to Peru and developed Carrion’s disease-like symptoms. Hence, this study aims to report on bacterial isolates from patients diagnosed with Carrion’s disease, which were found to be caused by *B. rochalimae* rather than *B. bacilliformis*, and to characterize the genomic aspects of *B. rochalimae* as a human pathogen. Five strains of *B. rochalimae* were identified using pangenomic and phylogenetic analysis. Additional analyses included core and clade-specific genes, gene ontology (GO), virulence factors (VF), and subcellular localization. This study identified five *B. rochalimae* strains from the regions of Ancash, Cajamarca, and Huanuco in Peru, suggesting regional circulation. The findings emphasize the importance of further research on *B. rochalimae* gene functions and its role in outbreak developments, highlighting the importance of improved diagnostics and enhanced surveillance.

## Summary

Carrion’s disease is a bacterial infection caused by *Bartonella bacilliformis*, characterized by fever and anemia in the acute phase and skin lesions in the chronic phase. However, a case with symptoms similar to Carrion’s disease has been reported, where *Bartonella rochalimae* was identified as the causative agent. Five *B. rochalimae* strains were isolated from human samples (one from Ancash, one from Cajamarca, and three from Huanuco). Phylogenetic and genetic analyses suggest that dogs could be part of the circulation of *B. rochalimae*, and that this bacterium may play a role in the development of outbreaks.

## Introduction

The *Bartonella* genus includes over twenty species, most of which are transmitted by arthropods and possibly eleven species have been associated with human diseases, however, for some *Bartonella* species, currently, specific vectors or hosts have not yet been identified [1]. The most relevant causative agents of human disease are *Bartonella rochalimae*, *Bartonella henselae*, *Bartonella quintana*, and *Bartonella bacilliformis* [2]. *B. rochalimae*, a Gram-negative pleomorphic bacterium, was first isolated in 2007 from a whole-blood sample from a 43-year-old American woman who traveled to Peru [2]. This patient had visited the Sacred Valley of Urubamba-Cusco and the Amazon Basin near Iquitos, additionally, she reported mosquito bites on her arms and legs [2]. After visiting Peru, the 43-years-old patient displayed Carrion’s disease-like symptoms, including fever, macular rash, enlarged spleen and anemia [2].

Carrion’s disease is a biphasic disease (includes Oroya fever and Peruvian wart) caused by *B. bacilliformis* transmitted by *Lutzomyia* sp. (sandflies), this disease is endemic to the Andean region in South American countries, however, cases were reported only in Peru in the last decades [3]. After the pandemic, the Ministry of Health of Peru reported an increase in cases in 2024, with peaks between February and May, reaching 299 cases, compared to just 17 cases reported the previous year [4]. The Oroya fever, acute phase, presents a severe hemolytic anemia due to the intense lysis of red blood cells, afterward, warts or eruptive lesions occur because of the proliferation of endothelial cells during the chronic phase; asymptomatic patients were also reported [3,5]. Despite *B. bacilliformis* being the only bacterium recognized as the agent of Carrion’s disease, other species has been associated with similar clinical manifestations, such as *B. rochalimae* and *Bartonella anchasensis* [2,6].

The diagnosis of the first documented case presenting Carrion’s disease-like symptoms caused by *B. rochalimae* followed a comprehensive laboratory approach [2]. Blood samples were cultured, and the resulting isolates were analyzed using transmission electron microscopy. Additionally, serum samples were tested for IgM and IgG detection [2]. Based on phenotypic characteristics, the bacterium was initially identified as a presumptive *Bartonella* species [2]. This preliminary identification was subsequently confirmed through molecular analysis of four gene sequences, which established the pathogen as a novel Bartonella species phylogenetically related to *B. clarridgeiae*, later designated as *B. rochalimae* [2].

The former clinical case is the first description of *B. rochalimae* as a human pathogen, indicating that the patient was an accidental host, in contrast, raccoons and foxes are recognized as natural reservoirs [2,7]. However, in 2024, a new case of a human infection by *B. rochalimae* was reported in which the patient developed endocarditis without fever, the patient had a congenital heart disease and reported contact with a dog [8]. Additionally, *B. rochalimae* was isolated from fleas (*Pulex irritans*) collected from domestic animals (cats and dogs) in three Peruvian regions, highlighting that *P. irritans*, known as human fleas, can be a vector [7]. Hence, this study aims to report on bacterial isolates from patients diagnosed with Carrion’s disease, which were found to be caused by *B. rochalimae* rather than *B. bacilliformis*, and to characterize the genomic aspects of *B. rochalimae* as a human pathogen.

## Materials and methods

### Bacterial isolated and growth

This study focuses on the analysis of strains identified as *B.rochalimae*. The selection of these specific strains was based on samples from the biological sample bank of the Laboratory of Vector-Borne Bacterial Diseases (LRNMEZOB) at the Instituto Nacional de Salud (INS), Peru, which maintains a collection of bacterial isolates from patients diagnosed with Carrion’s disease. This repository comprises 200 isolates collected between 2005 and 2019.

The sample size for genome sequencing was determined using the OpenEpi tool. A total of 155 bacterial isolates were randomly selected through stratified sampling, proportional to the number of strains isolated in each region. The use of these isolates was authorized by the Director of the National Center for Public Health at INS.

All the 155 isolates were reactivated in a biphasic medium, consisting of a solid phase made of Columbia agar (OXOID, UK), 0.25% yeast extract (OXOID, UK), and 10% defibrinated sheep blood, and a liquid phase of RPMI 1640 supplemented with L-glutamine and sodium bicarbonate (Gibco, UK). The cultures were incubated at 28°C for 4–5 days. Subsequently, the colonies were harvested using the liquid phase and 0.5 mL was inoculated into agar plates containing Columbia agar (OXOID, UK), 0.25% yeast extract (Gibco, Paisley, UK), and 10% defibrinated sheep blood, and finally, the plates were incubated at 28°C until colonies visualization.

The laboratory and epidemiological data were retrieved from the Laboratory Information System NETLAB V1.0 and epidemiological records. All data associated with *B. rochalimae* were summarized. LRNMEZOB received only patient samples and did not conduct any medical evaluations. However, all health centers are required to comply with the Peruvian technical standard when submitting diagnostic requests for Carrion’s Disease [9]. During the outbreaks of this diseases, samples may also be collected from asymptomatic individuals with relevant epidemiological backgrounds [9].

### DNA extraction and whole genome sequencing

Genomic DNA was extracted using a GeneJet NGS Cleanup Kit (Thermo Fisher Scientific, Lithuania), following the manufacturer’s instructions. DNA quality and concentration were assessed with the Qubit fluorometer (Thermo Fisher, USA). The *B. bacilliformis* isolates were initially confirmed through PCR amplification of the ialB gene (F: CACCATGAAAAAAATATTAAATTTATTTG and R: TTTTTGCAAAGAAGTTAAACGCTTAAG). For samples yielding negative results for the ialB gene, the internal transcribed spacer (ITS) region was amplified using nested PCR as a secondary confirmation (F_external_: CAATGGCGCGGTTAAGCTGCCAATC, R_external_: CTCTTTCTTCAGATGATGATCCC, F_internal_: CTTTGAGCTCTTCCTTGCGA, and R_internal_: GCCTGTTCTATTGAAATCGTG), also with primers specifically designed for this study.

Short read genome sequencing was performed on 155 strains using Illumina NovaSeq 6000 System S4(Illumina, USA). Paired-end genomic libraries (2 x 150 bp) were prepared with the NextEra DNA library preparation kit (Illumina, San Diego, CA). However, only non-*B. bacilliformis* strains were included in this study.

### Data quality control and genome assembly

Sequencing quality was assessed employing FASTQC v0.11.9 tool [10], reads were processed with Trimmomatic v0.39 (SLIDINGWINDOW:4:20 MINLEN:149) [11], and KRAKEN2 v0.11.9 was used for a taxonomic sequence classification [12]. The *de-novo* assembly was performed using SPADES v0.11.9 [13], genome assemblies (number of aligned reads, genome completeness, GC content) were evaluated with QUAST v0.11.9 [14] using the genome reference *B. rochalimae* strain ATCC BAA-1498 (GenBank access: GCF_000706645.1), depth of coverage was analyzed using QUALIMAP v0.11.9 [15].

### Identification of *B. rochalimae* specimens

Identification and description of the *Bartonella* genomes was performed using 326 genomic assemblies of *Bartonella* genus retrieved from National Center for Biotechnology Information database (NCBI).

We carried out a pangenomic analysis using ROARY software v0.11.9 (-cd: 80%, -i: 90%) on the annotated files in gff format (containing all the coding sequences or CDS) obtained using PROKKA v0.11.9 tool [16,17]. An exploratory phylogenetic analysis was performed based on matrix of single nucleotide polymorphism (SNP) using SNP-sites software [18]. A phylogenetic tree was constructed using maximum-likelihood algorithm with 1000 bootstrap replicates, employing RAxML program v8.2.10 [19], the consensus tree was rooted at the midpoint in Figtree v0.11.9 [20] and inspected in microreact (microreact.org) [21].

Genomes seemingly related to ATCC BAA-1498 B. *rochalimae* reference strain (including those labelled as *Bartonella* sp.) were visually identified and were submitted to Ortho-ANI distance estimations, using a species cut-off of 95–96% with the OAT software [22]. Additionally, a heatmap was generated from these distances using R packages: igraph, reshape2, scales, and ggplot2. A secondary pangenomic analysis was performed following the previous established pipeline, with the only modification being the use of RAxML configured for 20 independent searches and 1,000 bootstrap replicates. Potential clades corresponding to metadata columns were further examined using microreact [21].

### Genes characterization

Core genes and clade specific coding sequences (CDS) were identified by a specific designed function in R (R v0.11.9), using the “presence-absence” roary output file of the second pangenomic analysis. Three features were estimated on the translated “pan_genome_reference” roary output file: 1) The gene ontology (GO) using eggNOG-mapper v2 [23]; 2) The virulence factors (VF) identified by comparing estimated coding sequences with the virulence factors database (VFDB) [24] using BLASTp [25] with a cutoff e-value 1e-7 and both 60% of subject and query coverages to exclude distant homologs, and 3) The subcellular localization with BUSCA server by setting “Gram-negative” as taxonomic origin option [26].

The analysis for clade and strains included gene counts by GO categories, subcellular localization. To identify the most relevant discriminatory GO categories between clades, a principal component analysis (PCA) was performed with the R package “vegan”, the most discriminant features, and the R package “ggplot 2”.

### Total-evidence phylogenetic analysis

*B. rochalimae* gene sequences (16s, ftsZ, gltA, groEL, rpoB, ssrA, and internal transcribed spacer 16s-23s) were retrieved from NCBI, those genes were compared into the similar regions of the genomes related to ATCC BAA-1498. Therefore, the MAFFT v7.525 program was then used to align the sequences [27], and SequenceMatrix was employed in the concatenation process [28]. The results were employed to create a “total evidence phylogenetic tree” using the maximum likelihood algorithm with 1000 bootstrap replicates [19], the tree was rooted at the midpoint in FigTree v0.11.9 and inspected in microreact [21].

## Results

### *B. rochalimae* isolated from patient with Carrion’s diseases diagnosis

The genomic characterization was performed using a minimum of approximately 5.5 million trimmed reads per strain, representing approximately 84% of the raw data, and with a GC content of approximately 35.68% (S1 Table). Sequence analysis was conducted on the isolates from patients diagnosed with Carrion’s diseases diagnosis, cause by *B. bacilliformis*. However, specie identification reveals five strains related to *B. rochalimae*. The genomes and raw sequencing data are available under BioProject ID PRJNA1209094. The genome accession numbers are: JBMUSJ000000000, JBMUSK000000000, JBMUSSL000000000, JBMUSM000000000, and JBMUSN000000000. The corresponding FASTQ files can be accessed through the following SRA accession numbers: SRR33039478, SRR33039479, SRR33039480, SRR33039481, and SRR33039482.

**Fig 1:**
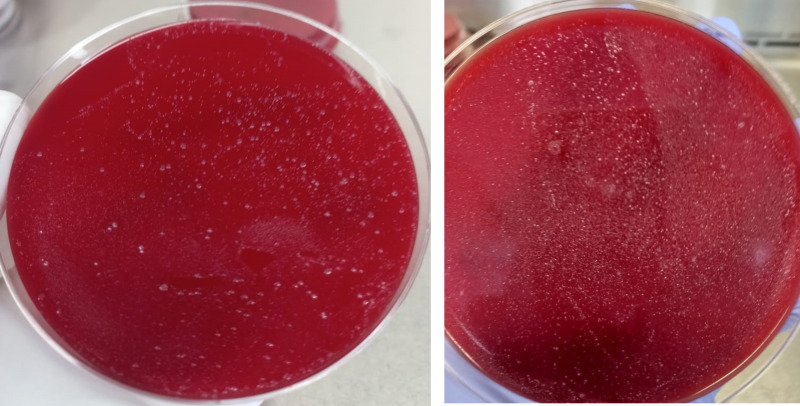
Colony Morphology of *B. rochalimae* on blood agar plates.

The bacterial cultures, grown on agar plates, did not exhibit distinct colony characteristics compared to *B. bacilliformis*. They appeared as small, round colonies with variable morphology and size, displaying confluent growth, regular borders, and a frost-like sheen when exposed to light (Fig 1).

As part of the protocol, all samples were evaluated by PCR using primers for the *ialB* gene and the ITS region. For all five strains, *ialB* detection was negative, while the ITS region was positive (200pb, compared to 300pb for *B. bacilliformis*).

These five *B. rochalimae* strains were isolated from endemic areas of Carrion’s disease in Peru: one from Ancash, one from Cajamarca, and three from Huanuco (Table 1). All strains were obtained from female patients; however, none presented similar clinical manifestations. Based on the main diagnostic tests used at LRNMEZOB-blood smear, bacterial culture, and, since 2016, an enzyme-linked immunosorbent assay (ELISA) developed using whole-soluble *B. bacilliformis* proteins as antigens [29] —ELISA and blood smear exhibit lower sensitivity for detecting *Bartonella rochalimae* compared to bacterial culture, as shown in this study. The used ELISA assay has been reported to exhibit high sensitivity (IgG 93.3% and IgM 90.0%) but relatively low specificity (IgG 88% and IgM 84%), which may lead to cross-reactivity with infections caused by other *Bartonella* species [29].

**Table 1:** Data on *B. rochalimae* strains and clinical, demographic, and diagnostic information of patients in which it was identified.

Strain	BioSample_Accession NCBI	Year of isolation	Region	Province	Distrit	Town	Age	Gender	Clinical manifestation	ELISA	Blood smear
Br-94-INS	SAMN46205632	2015	Ancash	Sihuas	Sihuas	Sihuas	31	Female	..	**IgM:** ··	..
										**IgG:** Positive	
Br-131-INS	SAMN46205633	2016	Huanuco	Huacaybamba	Conchabamba	Pachachin	9	Female	Jaundice	**IgM:** Negative	Negative
										**IgG:** Positive	
Br-132-INS	SAMN46205634	2016	Huanuco	Huacaybamba	Pinra	Huaracillo	14	Female	Headache, arthralgia	**IgM:** Negative	Negative
										**IgG:** Negative	
Br-136-INS	SAMN46205635	2007	Cajamarca	Chota	..	..	9	Female	..	**IgM:** ··	Negative
										**IgG:** ··	
Br-174-INS	SAMN46205636	2016	Huanuco	Huacaybamba	Conchabamba	Pachachin	17	Female	Fever, pallor, headache, general discomfort, jaundice, skin lesions, hyporexia	**IgM:** Negative	Negative
										**IgG:** Negative	

(··) No Data

**Fig 2:**
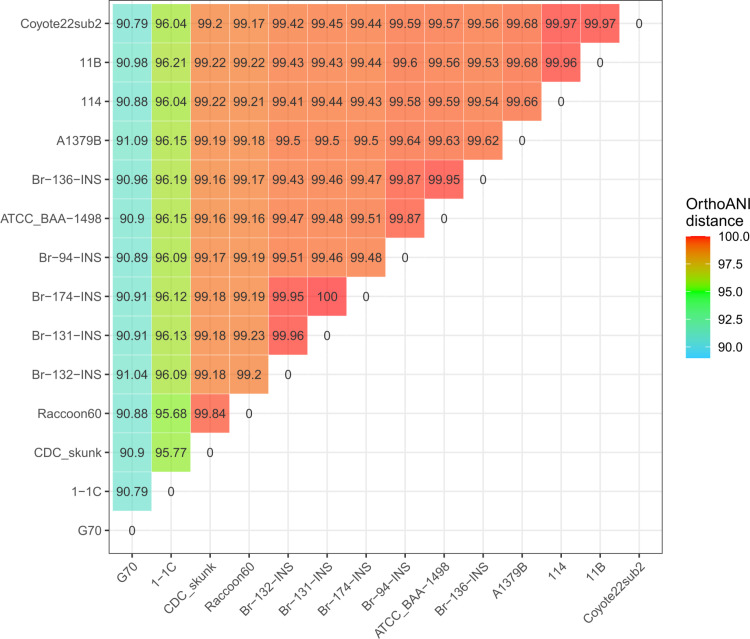
Heatmap of ortho-ANI distances, including patient-isolated strains of this study (Br-94-INS; Br-131-INS; Br-132-INS; Br-136-INS; Br-174-INS).

The phylogenetic analysis includes the five *B rochalimae* strains isolated from human and eight B. *rochalimae* strains identified from retrieved genomes of the NCBI, only labelled as “*Bartonella* sp”. The results of the ortho-ANI analysis (Fig 2) show the strain G70 (GCA_023500045.1), with distances from 0.901 to 0.905, could not be considered as a *B. rochalimae* member. Conversely, the remaining strains are related to ATCC BAA-1498, including the five isolates obtained from patients with CD-like symptoms and the remaining seven sequences retrieved from NCBI.

In addition, the seven sequences were recovered from blood samples of different mammals: 1) isolate 1-1C (GCA_002810325.1) is related to the “brown rat” (*Rattus norvegicus*); 2) isolate CDC skunk (GCA_002022545.1) and isolate Racoon60 (GCA_002022585.1) were related to the “striped skunk” (*Mephitis mephitis*) and “raccoon” (*Procyon lotor*), respectively; 3) isolate 114 (GCA_002022645.1), isolate coyote22sub2 (GCA_002022565.1), isolate A1379B (GCA_002022485.1) isolate 11B (GCA_002022625.1) belonged to canids (*Canis lupus*, *Canis latrans*, *Vulpes vulpes*, and *Urocyon cinereoargenteus*).

**Fig 3:**
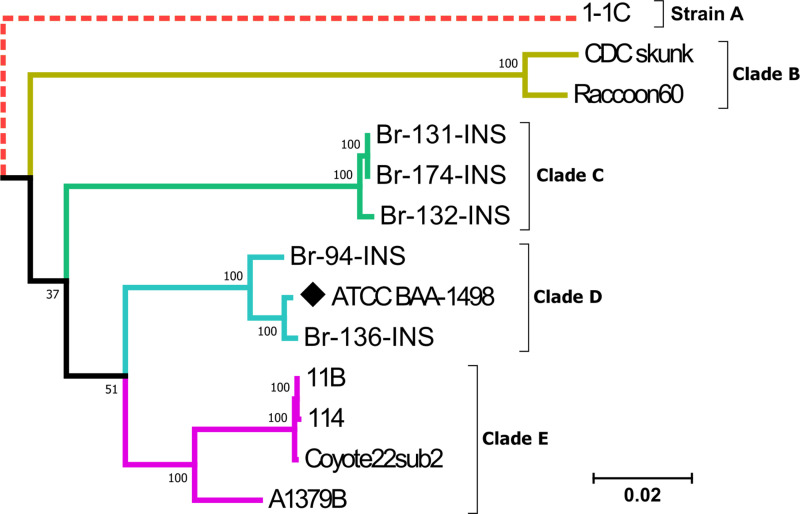
SNP-based RAxML Maximum Likelihood (ML) phylogenetic tree of *B. rochalimae* with bootstrap node support. The tree includes strains isolated from patients (Br-94-INS; Br-131-INS; Br-132-INS; Br-136-INS; Br-174-INS) and depicts strains and clades associated with *B. rochalimae*. It features a distant strain (A), represented by a red dotted branch (not to scale), and four clades: B (olive branches), C (green branches), D (light blue branches), and E (pink branches). To improve visualization, the branch length of strain A was compressed. The ATCC BAA-1498 strain is indicated by a black diamond.

The maximum likelihood phylogenetic tree allows the identification of one distant strain and four clades, each one shows an association with a specific host. Strain A is derived from “brown rat”, clade b includes strains from “striped skunk” and “raccoon”, clades C and D are made up of human samples (when strain ATCC BAA-1498 was localized in clade D), and clade E includes strains from canids. Bootstrap supports are 100; however, only node support of clades D and E resulted with a bootstrap value of 51 (Fig 3).

### Gene Ontology, virulence factors and subcellular localization

**Fig 4:**
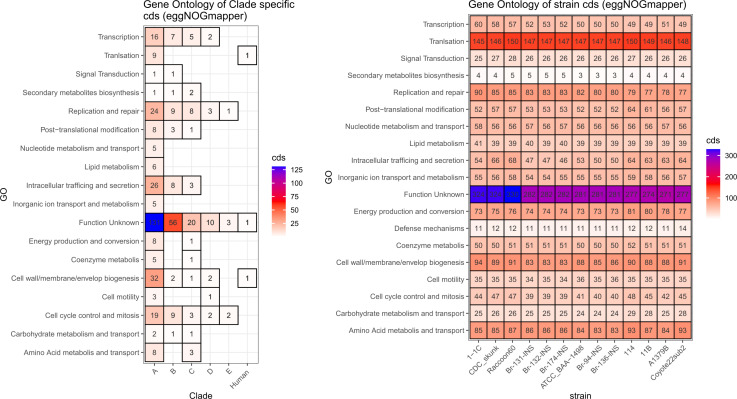
Comparison of functional annotations in coding sequences (CDS) obtained by GO, compared across clades (left) and strains (right), including patient-isolated strains of this study (Br-94-INS; Br-131-INS; Br-132-INS; Br-136-INS; Br-174-INS). Each cell reflects the number of unique CDS found in a particular strain or clade.

The Gene Ontology (GO) displays 18 functional annotations in all the sequences, Fig 4, the results were compared among clades and strains, showing the largest number of genes remains with unknown function. Likewise, the number of genes related to each functional annotation was similar, at the strain level.

Additionally, a human clade was included in the analysis which consists of the ATCC strain, and the isolates obtained from patients. This clade allows the identification of one common gene for two functions (translation and cell wall/membrane/envelop biogenesis) and one unknown function.

**Fig 5:**
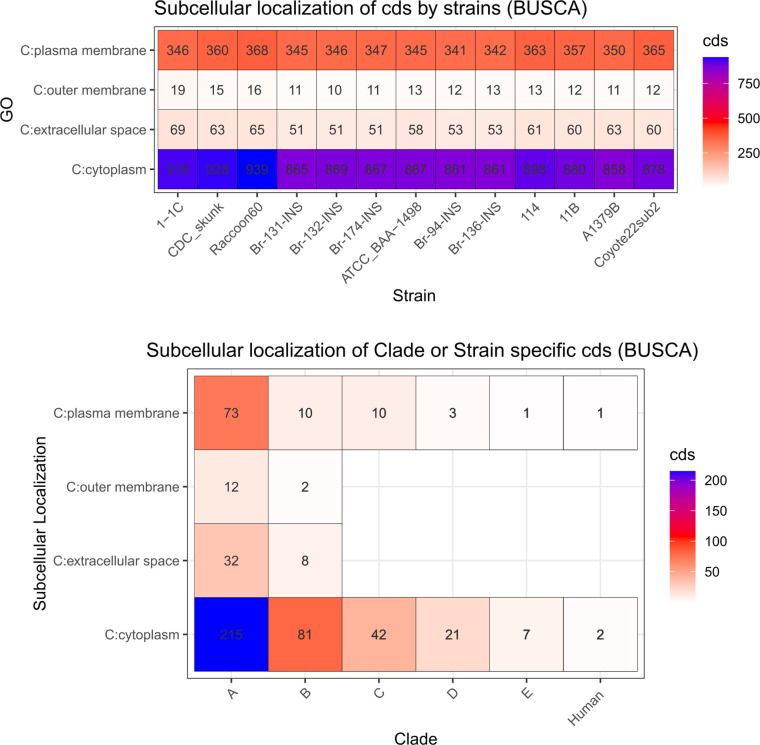
Comparison of subcellular localization in coding sequences (CDSs) predicted by BUSCA across clades (Bottom) and strains (Top), including patient-isolated strains from this study (Br-94-INS; Br-131-INS; Br-132-INS; Br-136-INS; Br-174-INS). Each cell indicates the number of unique CDSs identified in a particular strain or clade.

An analysis to obtained subcellular localization was also performed (Fig 5). At strain level, the number of subcellular localizations remains similar. Whilst human-derived-isolates are shares features with clade C, D and E.

**Fig 6:**
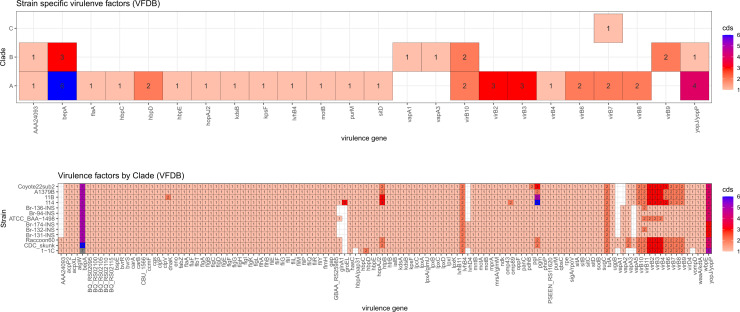
Comparison of virulence factors in coding sequences (CDSs) identified using VFDB across across strains (bottom) and clades (top), including patient-isolated strains from this study (Br-94-INS, Br-131-INS, Br-132-INS, Br-136-INS, and Br-174-INS). Each cell represents the number of unique CDSs found in a particular strain or clade.

Furthermore, differential genes at species level and virulence factors were identified in each *B. rochalimae* strain, the results were also supported by clade-specific analysis which provides differential genes among them, Fig 6 and Table 2. The quantity of the virulence factors is similar among strains, but there are unique differences in human-isolated strains. The AAA24093, glnA1, ugpB, and vapA3 were missing in the five isolates from patients and ATCC BAA-1498, also, Br-131-INS, Br-132-INS, and Br-174-INS were the only strains containing lvhB4 factor as in Raccoon60 and CDC_skunk.

**Table 2:** Differential genes of *B. rochalimae.*

Gene Ontology	Description
Intracellular trafficing and secretion	Conjugal transfer protein TraG
	Classified as an adhesion factor
	The Type IV secretion system (T4SSs)
	Preprotein translocase component
	Preprotein translocase subunit
	The Sec-independent protein translocase protein
	Major outer membrane lipoprotein
inorganic ion transport and metabolism	Membrane proteins
	Permeases
	ABC transporters
	cationic/protonic
Cell cycle control and mitosis	Peptide synthesis, septal ring localization
	Chromosome segregation
	Membrane assembly
	Bacterial sex pilus assembly
Transcription	Type II toxin-antitoxin system
	DNA-directed RNA polymerase
	AntA/AntB antirepressor
	transcriptional regulatory protein
Replication and repair	ATP-dependent DNA helicases
	Chromosomal replication initiator protein
	Crossover junction endodeoxyribonuclease
	Lambda exonuclease family protein
	Phage-related integrase
Energy production and conversion	ATP synthase epsilon chain
	Cytochrome bo[3] ubiquinol oxidase
	NADH-quinone oxidoreductase
	Succinate dehydrogenase

**Fig 7:**
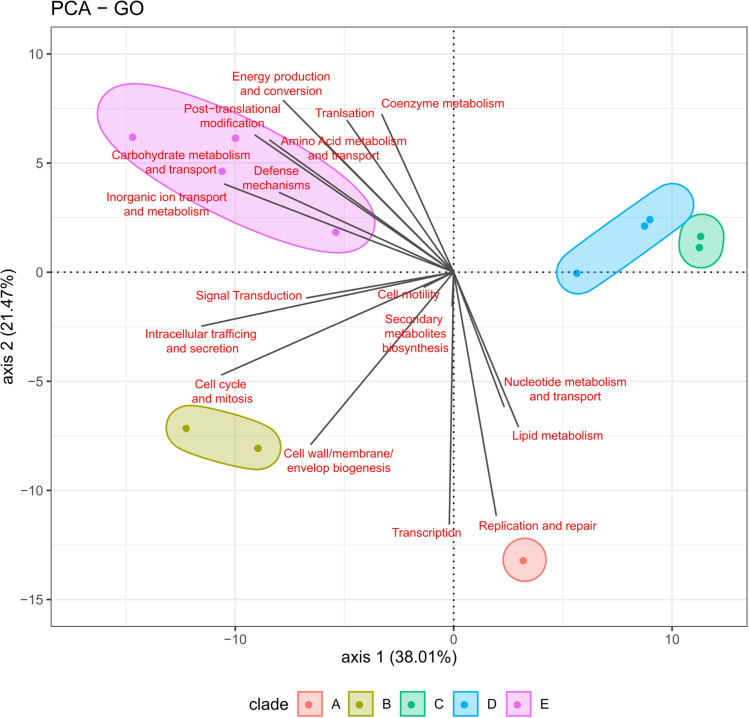
Principal Component Analysis based on GO results, show per strain. Arrow lengths are proportional to category contribution on both axes.

Finally, the PCA for functional annotation pointed out the first component (PC1) accounted for the 38.01% of the variation and the second component (PC2) accounted for the 21.47% of the variation, (p-value<0.001) (Fig 7). The PC1 includes “Intracellular trafficking and secretion”, “inorganic ion transport and metabolism” and “Cell cycle control and mitosis”, and the PC2 includes “Transcription,” “Replication and repair” and “Energy production and conversion”. Likewise, the results show isolates are gathered based on the presence and quantity of the genes, and the relation obtained by the GO analysis.

According to the GO at the PC1 component, some of the elements were previously identified as *Bartonella*-related proteins. The “Intracellular trafficking and secretion” includes a BadA protein classified as an adhesion factor identified in *B. quintana*, and the proteins of the Type IV secretion system (T4SSs) and the effector delivery system VirB2 – VirB11 characterized in *B. henselae*, *B. quintana*, and *B. tribocorum*. In the “Cell cycle control and mitosis” element, the protein bepA is identified as a virulence factor in *B. henselae* and has unique variants in the strain A, and clades B, C, and D.

### Phylogenetic relationship between strains isolated from humans and other mammals

**Fig 8:**
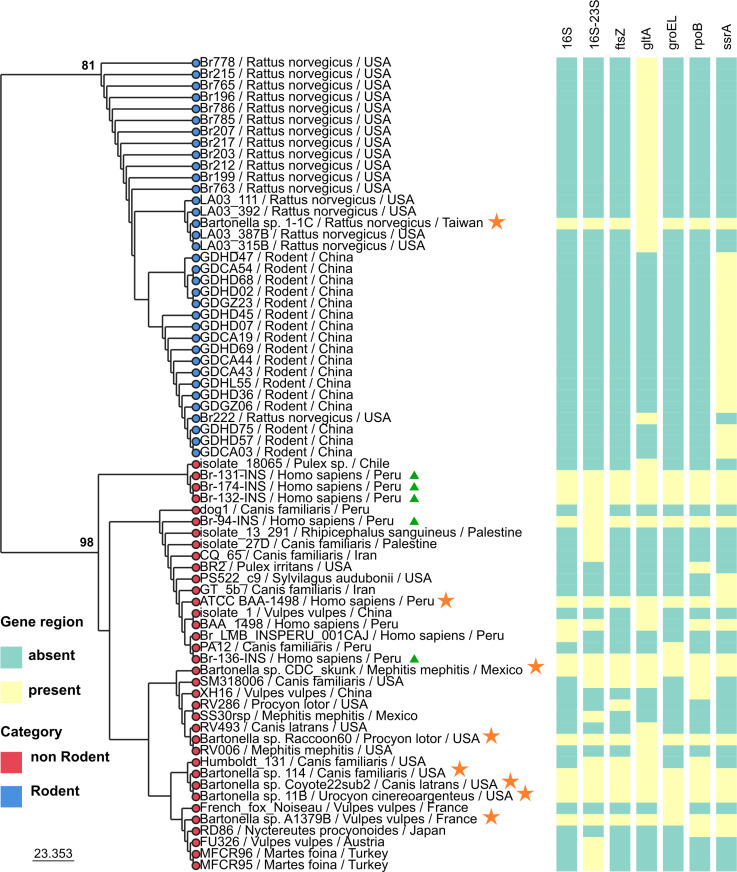
Total evidence Maximum Likelihood phylogenetic tree constructed using a partial matrix of seven regions (16S, 16S-23S, ftsZ, gltA, groEL, rpoB and ssrA). Bootstrapsupports values ≥ 50 are indicated above major branches. The tree distinguishes between Rodent clade (blue circles) and non-rondent clade (red circles). The presence or absence of each region is indicated by yellow and light blue squares. Genomes generated in this study are highlighted with triangles, while genomes downloaded from NCBI are marked with stars. Tip labels include the strain name, host, and country of origin.

The resulting total evidence phylogenetic tree displayed two major clades (Fig 8): a "rodent clade" (BS = 81), named after *Rattus norvegicus* and other unspecified rodent species; and a "non-rodent clade" (BS = 98), associated with humans, canids (*Canis familiaris, Canis latrans, Vulpes vulpes, Urocyon cinereoargenteus*), other canidae members (*Nyctereutes procyonoides*), mustelidae (*Martes foina*), skunk (*Mephitis mephitis*), raccons (*Procyon lotor*), rabbits (*Sylvilagus auduobonii*) and blood-feeding arthropods (*Rhipicephalus sanguineus, Pulex irritans*).

## Discussion

Over the past century, Carrion’s disease has been consistently linked to *B. bacilliformis*, with clinical manifestations typically characterized by fever and anemia in the acute phase and skin lesions in the chronic phase [3,5]. Consequently, all patients from Peruvian endemic areas presenting these symptoms have been evaluated as potential cases of Carrion’s disease. So, the confirmatory diagnostic strategies for this diseases aims to evaluate presence of *B. bacilliformis* [30], and the amplification of specific genomic region are used to detect this bacterium in blood samples [31,32].

However, over time, *B. rochalimae* and *B. ancashensis* have also been reported as presumptive pathogens causing Carrion’s disease-like symptoms [2,33], these findings support our results indicating that other species within the *Bartonella* genus can produce symptoms similar to Carrion´s disease (Table 1–3). In this study, all the samples collected from the biobank were isolated from patients diagnosed with Carrion´s disease, so the expected results were always to find *B. bacilliformis*. Therefore, the identification of five *B. rochalimae* strains highlights the need to enhance molecular confirmatory diagnostics and adapt the surveillance system to detect other Bartonella species, especially in cases where strains are isolated from patients with negative IgM results by ELISA and blood smear.

The five *B. rochalimae* strains—one from a reported Carrion’s Disease outbreak in Cajamarca in 2007, one from active surveillance of Carrion’s Disease in 2015, and three from a separate Carrion’s Disease outbreak in Huanuco in 2016 (Table 1)—suggest the potential circulation of *B. rochalimae* across different regions of Peru, previously reported as endemic. Notably, Ancash, located 300 km from Huanuco and 600 km from Cajamarca, is an area where *B. bacilliformis*, B*. ancashensis*, and *B. rochalimae* have been documented [6]. Further data on migration patterns between endemic areas could provide additional insights into the pathogen’s spatial distribution. Finally, considering that the samples were collected during outbreaks of Carrion’s Disease, it is important to emphasize the public health risk associated with this pathogen.

Further analysis reveals a lack of information about the function of genes from *B. rochalimae* (Figs 4 and 7), with the subcellular localization of most of the coding sequences identified in the plasma membrane and cytoplasm (Fig 5). The significant portion of protein with unknown functions highlights *B. rochalimae* as an overlooked pathogen, indicating that either the three-dimensional (3D) structural information is unavailable or no homologous proteins have been found [34]. Although protein conservation depends on the evolutionary information of the species, we believe the information gap requires more support at both the genus and species levels [34,35]. Ergo, understanding the structural characteristics of specific proteins is crucial for comprehending the molecular mechanisms of infection and developing therapeutic strategies against pathogens [34,35].

Likewise, the analysis of virulence factors shows a clear difference between human isolate strains and those from other mammals (Fig 6). The absence of AAA24093 endotoxin gene [36], glnA1 glutamylamine synthetase gene [37], ugpB chaperone gene to prevent bile‐induced aggregation [38], and vapA3 gene for intracellular replication [39] in all patient strains and ATCC BAA-1498 could be a crucial feature for specific human infection, as this similarity is consistent among them. Another important virulence feature is the lvhB4, reported as a factor in *Legionella* sp. that affects the infection capacity and is involved in persistence in environmental reservoirs [40]. This gene has been identified only in all *B. rochalimae* strains from Huanuco.

Finally, the phylogenetic tree based on total evidence reveals two clades (Fig 8). All patient strains (Br-94-INS; Br-131-INS; Br-132-INS; Br-136-INS; Br-174-INS) were grouped into clade 2, along with strains isolated from canids. This grouping suggests that canids could play a role in the circulation of *B. rochalimae* to humans, although further studies are required to confirm this hypothesis. This hypothesis is supported by the fact that strains from blood-sucking arthropods, commonly ectoparasites of canids, also belong to this clade. Additionally, this aligns with data from Diniz et al., who reported *B. rochalimae* infections in dogs (*Canis familiaris*) [41], as well as from Yore et al., who found *B. rochalimae* infections in dogs and fleas during a prevalence study at a dog shelter in Florida, suggesting that flea-infested dogs may serve as a reservoir host for *B. rochalimae* [42]. However, a broader scope must be considered, as this pathogen has also been reported in *Pulex simulans* collected from wild animals (*Mephitis macroura*) [43].

## Conclusion

This study provides new insights into the genomic landscape of *B. rochalimae*, an emerging human pathogen associated with Carrion’s disease. Our analysis identified five strains of *B. rochalimae* from Peruvian endemic areas to Carrion’s disease (Ancash, Cajamarca and Huanuco), confirming the circulation of this pathogen and suggesting a key role for canids in its circulation. The genomic data underscore a notable gap in our understanding of the functional roles of many B*. rochalimae* genes, highlighting the need for further investigation into their specific contributions to pathogenicity.

The absence of certain virulence factors in human isolates, coupled with the distinctive phylogenetic clustering of strains from different hosts, which could suggest that *B. rochalimae* may exhibit host-specific adaptations. These findings emphasize the importance of developing more refined diagnostic tools and enhancing surveillance systems to accurately identify and monitor *B. rochalimae*. Given the observed genomic diversity and potential for regional spread, further research into the epidemiology and virulence of *B. rochalimae* is essential for improving public health responses and mitigating the impact of this pathogen.

In summary, this study highlights the potential pathogenic role of previously under-recognized *Bartonella* species, including those with different vectors or reservoirs. It provides crucial insights into the presence of *Bartonella rochalimae* in endemic areas of Carrion’s disease and suggests a possible involvement of canids in this process, however further studies are needed to confirm this hypothesis. The observed genetic variability and the absence of certain virulence factors in human isolates underscore the need for improved diagnostics and control strategies. Given the pathogen’s potential for regional spread, strengthening surveillance systems and outbreak response is essential. Furthermore, recognizing *B. rochalimae* as a potential cause of Carrion’s Disease or a similar syndrome would have significant implications for prevention and control efforts, as it likely relies on different transmission dynamics, unlike *B. bacilliformis*, which has no known animal reservoir. An integrated approach considering human, animal, and environmental health is vital for effective prevention and management.

## Supporting information

S1 Table Details of the date used by Illumina NovaSeq 600 System S4. (DOCX)
