# Supplementary material for: New insights into the genomic information of an overlooked human pathogen: Bartonella rochalimae causative agent of Carrion’s disease
Source: PLoS Negl Trop Dis. 2025 Apr 17;19(4):e0013040. doi: 10.1371/journal.pntd.0013040 (PMC12043232; doi:10.1371/journal.pntd.0013040)
Supplement: S1 Table — (DOCX) [file pntd.0013040.s001.docx]

**Supporting information**

S1 Table. Details of the date used by Illumina NovaSeq 600 System S4

| Sample | Br-94-INS | Br-131-INS | Br-132-INS | Br-136-INS | Br-174-INS |
| --- | --- | --- | --- | --- | --- |
| Total reads | 11 577 970 | 15 588 636 | 6 659 164 | 14 706 728 | 7 339 342 |
| Total trimmed reads | 10 158 854 | 13 573 014 | 5 791 344 | 12 475 082 | 6 402 626 |
| % Trimmed reads | 87.74 | 87.07 | 86.97 | 84.83 | 87.24 |
| Contigs (>= 1000 bp) | 21 | 18 | 20 | 17 | 19 |
| Largest Contig | 360 625 | 369 704 | 369 703 | 660 162 | 369 704 |
| % CG | 35.66 | 35.69 | 35.68 | 35.67 | 35.69 |
| % mapped reads | 100 | 98.6 | 99.74 | 100 | 99.94 |
| Genome fraction (%) | 98.54 | 96.70 | 96.61 | 98.71 | 96.55 |
| Avg. coverage depth | 1 156 | 1 528 | 659 | 1 466 | 730 |
